# A novel statistical test for treatment differences in clinical trials using a response-adaptive forward-looking Gittins Index Rule

**DOI:** 10.1111/biom.13581

**Published:** 2021-10-20

**Authors:** Helen Yvette Barnett, Sofía S. Villar, Helena Geys, Thomas Jaki

**Affiliations:** 1 MRC Biostatistics Unit, University of Cambridge, Cambridge, UK; 2 Janssen Pharmaceutica, Beerse, Belgium; 3 Medical and Pharmaceutical Statistics Research Unit, Lancaster University, Lancaster, UK

**Keywords:** allocation probability, inference, nonmyopic, power, testing for superiority

## Abstract

The most common objective for response-adaptive clinical trials is to seek to ensure that patients within a trial have a high chance of receiving the best treatment available by altering the chance of allocation on the basis of accumulating data. Approaches that yield good patient benefit properties suffer fromlow power from a frequentist perspective when testing for a treatment difference at the end of the study due to the high imbalance in treatment allocations. In this work we develop an alternative pairwise test for treatment difference on the basis of allocation probabilities of the covariate-adjusted response-adaptive randomization with forward-looking Gittins Index (CARA-FLGI) Rule for binary responses. The performance of the novel test is evaluated in simulations for two-armed studies and then its applications to multiarmed studies are illustrated. The proposed test has markedly improved power over the traditional Fisher exact test when this class of nonmyopic response adaptation is used. We also find that the test’s power is close to the power of a Fisher exact test under equal randomization.

## 1 Introduction

Equal randomization (ER) between treatment arms is the gold standard for any clinical trial (eg, Schulz, 1996), as such a randomization scheme will give the trial the highest power to detect a treatment difference (Pocock, 1979) under certain assumptions. While the purpose of any trial is to gain information about an experimental treatment, there is also the ethical consideration of the patients in the trial, and these two goals often conflict with one another. This has triggered the development of adaptively randomized trial designs, where the probability of a patient receiving a particular treatment is altered sequentially throughout the trial based on previous patients’ responses in order to treat subsequent patients on treatments that are believed to be superior. The use of such adaptive randomization has long been suggested for implementation in clinical trials for the advantages in patient benefit it offers (eg, Rosenberger and Lachin, 1993; Hu *et al*., 2006) and a vast methodological literature has been developed on the subject of how to update this patient allocation rule (eg, Rosenberger and Lachin, 2016; Williamson *et al*., 2017; Mozgunov and Jaki, 2020).

Multiarmed bandit models are the optimal idealized solution (in terms of patient benefit) to response-adaptive allocation (Gittins, 1979). While their original motivation was within trials, they have found wide application outside of trials (eg, Vermorel and Mohri, 2005; Gittins *et al*., 2011) but have, to our knowledge, not actually been used in the clinical trials setting. One of the reasons is that both the optimal solution and the computationally efficient approximate solution, the Gittins Index Rule (Gittins, 1979), is deterministic. In the clinical trials setting, the deterministic nature of the Gittins Index Rule for patient allocation and the assumption of infinite sample size are problematic due to the inherent risk of bias (Hardwick and Stout, 1991). Modifications of the Gittins Index Rule have been developed to apply the classic multiarmed bandit framework to deriving nearly optimal patient allocation procedure in clinical trials using adaptive randomization (Villar *et al*., 2015; Williamson and Villar, 2020). These modifications allow for randomization, consider finite-sized trials, and cater for patient accrual in blocks rather than individually. One particular modification of note is known as the modified forward-looking Gittins Index Rule (Villar *et al*., 2015), which offers the advantage that patient allocation is no longer deterministic. Instead, patient allocation is random according to an allocation probability. This probability can be calculated exactly or using MC simulations that themselves use the deterministic Gittins Index Rule.

Extending to adaptive randomization to adjust allocation probabilities according to covariates of patients is an important step in the area of personalized medicine. Adjusting for covariates or biomarkers not only allows for higher levels of patient benefit within the trial, but also for the targeting of experimental treatments to patient groups that will see the most rewards when the treatment is marketed. Villar and Rosenberger (2018) introduce a method that allows covariate adjustment, using a modified forward-looking Gittins Index Rule, henceforth referred to as the covariate-adjusted response-adaptive randomization with forward-looking Gittins index (CARA-FLGI) procedure.

Nonmyopic bandit-based procedures increase patient benefit by looking forward a considerable way into future patients’ allocations. Hence they do not have the shortcomings associated with myopic procedures that only take into account past information in the allocation of the present patient, namely, that in the exploring versus exploiting theory of allocation rules (Gittins *et al*., 2011), they do not explore enough. Consequently, such myopic procedures can settle too early on an inferior arm (Villar *et al*., 2015; Smith and Villar, 2018). Like all response-adaptive designs, they have the drawback that the resulting designs often lack in power due to substantial imbalance between patient groups.

The purpose of most response-adaptive trials is to identify superior treatments quickly, and in doing so, the resulting patient allocation favors the superior treatment. When directly performing inference on the binary responses of the patients to determine the outcome of the trial, there must be a trade-off between patient benefit and power. For a two-armed trial (under equipoise and equal variance assumptions), the closer the allocation is to equal sample size in each group, the higher the power but smaller the patient benefit. Likewise the further from equal the sample sizes between treatments can be, the larger the potential patient benefit but lower the power. Bandit-based designs provide high patient benefit, and therefore suffer from low power, which is a concern for their implementation (Villar *et al*., 2015). Previous work on response-adaptive randomization (RAR) has almost exclusively focused on the question of design while using traditional approaches for inference (eg, contingency table based for binary outcomes). In this work we develop an entirely novel perspective on the analysis of such trials. The idea is based on the observations that for bandit-based designs, when the patient allocation favors a treatment, this is an indication of the superiority of that treatment. It is therefore intuitive to use this indication to analyze results from a trial utilizing an FLGI procedure. In this paper we discuss how testing based on allocation probabilities from the CARA-FLGI procedure can be used as an alternative to testing based on the binary outcome of response to test the null hypothesis of no treatment effect difference. Alternative approaches to inference that are tailored to the specific RAR algorithm, such as the randomization test (Simon and Simon, 2011) have been applied to the FLGI design (Villar *et al*., 2018). Such an approach preserves type I error under broad assumptions but results in substantial power loss when compared to traditional inference in a fixed randomization trial. Motivated by the potential for a higher powered response-adaptive randomized trial due to the completely different nature of the sufficient statistics for the test (the test statistic being a function of allocation probabilities, which require the partial sums of successes and failures on each of the treatment arms after each block rather than the summary of these binary responses on each of the treatment arms at the end of the trial), this novel approach to decision making expands the potential of use for alternative methodologies in adaptive randomization. Our proposed methodology is exactly the solution to one of the key concerns posed in the National Science Foundation 2019 report, *Statistics at a Crossroads: Who Is for the Challenge?*, stating “A fundamental issue is the development of inference methods for post subgroup selection” (He *et al*., 2019).

In the following, Section 2 provides a review of the CARA-FLGI method, in particular how the allocation probabilities are calculated. Section 3 derives the testing procedure for the use of the allocation probabilities in such a trial. Section 4 then illustrates the use of the testing procedure in both a real multiarm trial scenario and simulations. Alongside this, its comparative properties and advantages over alternative methods are presented before we conclude with a discussion in Section 5.

## 2 Properties of Allocation Probabilities

### 2.1 The CARA-FLGI procedure

Throughout this paper, it is assumed that the CARA-FLGI procedure, as described by Villar and Rosenberger (2018), is applied. It is worth noting that although in the following we focus on the covariate-adjusted case, where there are multiple biomarker categories that partition the patient population labeled *z* = 1, … *n_z_*< ∞, the procedure of using allocation probabilities for inference purposes as we describe can indeed be applied to any FLGI allocation such as those presented by Villar *et al*. (2015); this is a strong advantage of the procedure. In fact, since the CARA-FLGI procedure reduces to the simpler FLGI allocation when there is only a single biomarker category, we also evaluate this case in the following work. It is worth strongly emphasizing that what we propose here is a novel testing procedure for a class of response-adaptive designs, not a novel response-adaptive design itself. The testing procedure can be used when interested in comparing an experimental arm (possibly out of many) to a control arm.

The trial set-up is as follows. Patients are accrued in blocks of prespecified size *B*, with total trial sample size *N* = *KB*, where *K* is the number of blocks. Biomarker categories that partition the patient population are defined, and each patient has an associated biomarker category. At the beginning of each block, an allocation probability is calculated for each biomarker category using the FLGI rule. This allocation probability is then used as the probability of assigning a patient within that biomarker category to the experimental treatment. This differs from using the standard Gittins Index Rule, as there is still randomness in the patient allocation. The larger the block size, the higher the randomization element, although this comes at the expense of deviating further from the optimality of patient benefit and computational cost. We consider the null hypothesis to be tested as *H*_0_ : No treatment effect difference in subgroup *z*, against the one-sided alternative *H_A_:* The experimental treatment is superior to the control in subgroup *z*.

Note that extensions to two-sided tests are straightforward. Traditionally, frequentist inference is carried out using statistical tests that are based on the observed success/failure outcomes from the trial. We propose however, to use the CARAFLGI allocation probabilities calculated at the beginning of each block to test these hypotheses. In the following, we provide an overview of how these probabilities are calculated using the FLGI in order to understand why they can be used for testing the hypotheses.

### 2.2 Calculation of CARA-FLGI allocation probability distribution

At the beginning of every block in the trial, the CARA-FLGI procedure calculates an allocation probability per treatment arm, per biomarker category. This FLGI procedure can use allocation probabilities calculated exactly (Villar *et al*., 2015), however the theoretical calculation is extremely intensive and therefore Monte Carlo (MC) simulations are often used to calculate these probabilities. In the following, we assume the use of MC simulations. For simplicity, we here assume that there are two treatment arms, labeled 0 for control and 1 for experimental, although the calculations are identical if multiple treatment arms are used. Let us recap how the procedure calculates the allocation probability for the experimental treatment for biomarker category *z, p*_alpro,z_, using MC simulations. It is worth noting that the following calculations are neither under the null nor alternative hypotheses as there is no assumption on treatment difference when calculating the probabilities themselves.

First, consider the current states of all biomarker categories at the beginning of the block, which are defined by the number of successes on the standard treatment (*s*_0,*z*_), failures on the standard treatment (*f*_0,*z*_), successes on the experimental treatment (*s*_1,*z*_), and failures on the experimental treatment (*f*_1,*z*_). We denote the current state *i* in category *z* by $S_{z}^{i}\left(s_{0,z}^{i},f_{0,z}^{i},s_{1,z}^{i},f_{1,z}^{i}\right)$ and the starting state for the block by $\begin{array}{l} S_{z}^{1}\left(s_{0,z}^{1},f_{0,z}^{1},s_{1,z}^{1},f_{1,z}^{1}\right)\text{.} \end{array}$ For the first block in the trial, this state is specified via an uninformative prior of $S_{Z}^{1}\left(1,1,1,1\right)\text{.}$. Although we advise an uninformative prior, an informative prior may be used if appropriate, provided the following distribution calculations are under the assumption of the same prior as used in the trial. From each of these category states, the procedure takes *n* MC runs labeled *j* = 1,…, *n*; each run is an independent block of the prespecified size *B*.

Within each MC run, *j*, the first patient is allocated to one of the treatment arms and success/failure is observed. The state for that biomarker category is updated, and the next patient is allocated to a treatment arm based on their (possibly updated) biomarker category state. This continues until the block is full, noting that patients in the same block may have different biomarker categories. This is repeated for each run, starting at the same initial states for each category.

The allocation of patients in the FLGI allocation procedure (and therefore in the MC simulations) depends on the Gittins Index (*GI*) Rule (Gittins, 1979). For a given patient, this rule takes the two available treatment arms (standard and experimental for the patient’s biomarker category) and calculates the *GI* for each arm. The patient is allocated to the treatment arm with the highest *GI*, breaking ties at random. At any given point, and consistent with the multiarmed bandit framework, we assume that patient success for a given treatment arm occurs according to the posterior success probability so far on that treatment arm. When FLGI probabilities are estimated through an MC procedure, the allocation probability for each category is the proportion of patients in each category allocated to the experimental treatment over the total number of runs.

This allocation probability is calculated (or approximated for large blocks) as $p_{\text{alpro,z}}=\sum_{j=1}^{n}Y_{j,z}/\sum_{j=1}^{n}X_{j,z}\text{.}$ Assuming that every biomarker category is equally likely to be observed in each block, $X_{j,z}∼\text{Bin}\left(B,\frac{1}{n_{z}}\right)$ is the number of patients belonging to biomarker category *z* (regardless of treatment) in the *j*th MC run, equalling *B* when *n_z_* = 1, and *Y_j,z_* is the number of patients in category *z* allocated to the experimental treatment on the *j*th MC run. For simplicity but with no loss of generality we assume $p\left(Z=z\right)=1/n_{z}\text{.}$

In order to calculate the distribution of *p*_alpro,z_, we consider the cumulative distribution function

$$F_{p_{\text{alpro}},z}\left(c\right)=P\left(p_{\text{alpro},z}\leq c\right)$$

for *c* ∈ [0,1], equivalent to

$$\begin{array}{lll} F_{P_{\text{alpro}}z}\left(c\right) & = & \mathbb{P}\left(\frac{\sum_{j=1}^{n}Y_{j,z}}{\sum_{j=1}^{n}X_{j,z}}\leq c\right)\\ & = & \mathbb{P}\left(\sum_{j=1}^{n}Y_{j,z}-c\sum_{j=1}^{n}X_{j,z}\leq 0\right)\text{.} \end{array}$$

Note that this assumes $\sum_{j=1}^{n}X_{j,z}>0.$ If no patients are incategory *z*, $\left(\sum_{j=1}^{n}X_{j,z}=0\right)\text{,}$ then the allocation probability is taken as 0.

The derivation of the discrete joint distribution of *X_j,z_* and *Y_j,z_* is provided in Web Appendix B in the online supporting information. For a given *j*, let the expectations be denoted as $E\left(X_{j,z}\right)=\mu_{x,z}$ and $E\left(Y_{j,z}\right)=\mu_{y,z}\text{.}$ and variances Var $\left(X_{j,z}\right)=\sigma_{x,z}^{2}$ and $\left(Y_{j,z}\right)=\sigma_{x,z}^{2}\text{.}$ The covariance of *X_j,z_* and *Y_j,z_* is given as

$$V_{z}=\sum_{x_{j,z},y_{j,z}}P\left(X_{j,z}=x_{j,z}\&Y_{j,z}=y_{j,z}\right)\times \left(x_{j,z}-\mu_{x,z}\right)\left(y_{j,z}-\mu_{y,z}\right)$$

where the sum is over all possible values of *x_j,z_* and *y_j,z_*. For $j\neq j^{\prime },\;\mathrm{cov}\left(X_{j,z},Y_{j^{\prime },z}\right)=0$ and therefore

$$\mathrm{cov}\left(\sum_{j=1}^{n}Y_{j,z},\;c\sum_{j=1}^{n}X_{j,z}\right)=cnV_{z}\text{.}$$

We approximate the following using the Central Limit Theorem for the MC runs: $\sum_{j=1}^{n}Y_{j,z}∼N\left(n\mu_{y,z}n\sigma_{y,z}^{2}\right),\;\sum_{j=1}^{n}X_{j,z}∼N\left(n\mu_{x,z},n\sigma_{x,z}^{2}\right)\text{,}$ and hence obtain the cumulative distribution function:

(1) $$F_{p_{\text{alpro}},z}\left(c\right)=\text{Φ}\left(\frac{n^{\frac{1}{2}}\left(c\mu_{x,z}-\mu_{y,z}\right)}{\sqrt{\sigma_{y,z}^{2}+c^{2}\sigma_{x,z}^{2}-2cV_{z}}}\right)\text{,}$$

with density function:

(2) $$f_{p_{\text{alpro}},z}\left(c\right)=\frac{n^{\frac{1}{2}}{\left(\mu_{x,z}\sigma_{y,z}^{2}+c\mu_{y,z}\sigma_{x,z}^{2}-\mu_{y,z}V_{z}-cV_{z}\mu_{x,z}\right)}^{\frac{3}{2}}}{\left(\sigma_{y,z}^{2}+c^{2}\sigma_{x,z}^{2}-2cV_{Z}\right)}\times \phi \left(\frac{n^{\frac{1}{2}}\left(c\mu_{x,z}-\mu_{y,z}\right)}{\sqrt{\sigma_{y,z}^{2}+c^{2}\sigma_{x,z}^{2}-2cV_{z}}}\right)\text{.}$$

Thus the distribution has a mode at $\mu_{y,z}/\mu_{x,z}\text{.}$ For a block starting at state $S_{z}^{1}\left(s_{0,z}^{1},f_{0,z}^{1},s_{1,z}^{1},f_{1,z}^{1}\right)\text{,}$ this is the ratio of the expected number of patients allocated to the experimental treatment in subgroup *z* compared to the total expected number of patients in subgroup *z*.

## 3 Testing for Superiority with Allocation Probabilities

We present the following theorem for testing for superiority using allocation probabilities.

**Theorem 3.1.** *Denote the true difference in success probability on the experimental treatment and control by* *p*_1_ − *p*_0_. *Consistently higher allocation probabilities for the experimental treatment, that is, more allocation probabilities greater than 0.5 at beginning of blocks within the trial, are observed if and only if* *p*_1_ − *p*_0_ > 0.

The theorem is a direct result of the following two lemmas, the proofs of which are given in Web Appendix C in the online supporting information.

**Lemma 3.1.** *For any state* $S_{z}^{i}\left(s_{0,z}^{i},f_{0,z}^{i},s_{1,z}^{i},f_{1,z}^{i}\right)$ with $P\left(p_{\text{alpro},z}<0.5\right)=\gamma \text{,}$ *its “mirror” state* $S_{z}^{i}\left(s_{1,z}^{i},f_{1,z}^{i},s_{0,z}^{i},f_{0,z}^{i}\right)$ *will give* $P\left(p_{\text{alpro},z}>0.5\right)=\gamma \text{.}$

**Lemma 3.2.** *Consistently higher allocation probabilities for arm 1 are a necessary and sufficient condition for* *p*_1_ − *p*_0_ > 0.

In order to use the allocation probabilities to test for superiority, we utilize the distribution of the allocation probabilities under the assumption that the treatment effects are equal (null distribution). The distribution of the allocation probability for a given block described by Equations (1) and (2) is calculated solely from the state at which that block starts. The success probabilities used in the CARA-FLGI are the posterior probabilities within the simulation, and not under any assumptions on the treatments themselves. However, the full null distribution of allocation probabilities for any block, *k*, is under the assumption that the treatment effects are equal. This distribution is a mixture distribution; a weighted sum of the allocation probability distributions $f_{p_{\text{alpro,}}\zeta ,z}$ from the potential states ζ, of which there are say *Z*, at the beginning of block *k*, with weights of probabilities of being in each of the potential states $P_{z}\left(\zeta \right)\text{,}$ for a given equal success probability for both treatments: $g^{\left(k\right)}\left(c\right)=\sum_{\zeta =1}^{Z}f_{p_{\text{alpro}},\zeta ,z}\left(c\right)P_{z}\left(\zeta \right)\text{.}$ This mixture distribution has point masses at 0 and 1, as for certain extreme states, the probability of allocating a patient to the experimental treatment is either 0 or 1. As this can occur fairly frequently, the resulting distribution can often not be used to formulate a nonrandomized level-*α*-test (see Fig. 3 in Smith and Villar, 2018, for a similar issue in a different setting). To overcome this bimodality problem we instead consider the number of blocks for which the allocation probability exceeds 0.5.

To achieve this, the allocation probabilities are dichotomized according to whether they are greater than 0.5. We then denote the binary outcome *α_k_* as 1 if the allocation probability to the experimental arm for block *k* is greater than 0.5 and 0 otherwise. Our test statistic, $Q=\sum_{k=1}^{K}\alpha_{k}\text{,}$ is then the total number of blocks for which the allocation probability to the experimental arm is larger then 0.5. Note that the value of 0.5 is for the two-arm setting. For a trial with multiple arms, this is the reciprocal of the number of arms.

**Figure 1:**
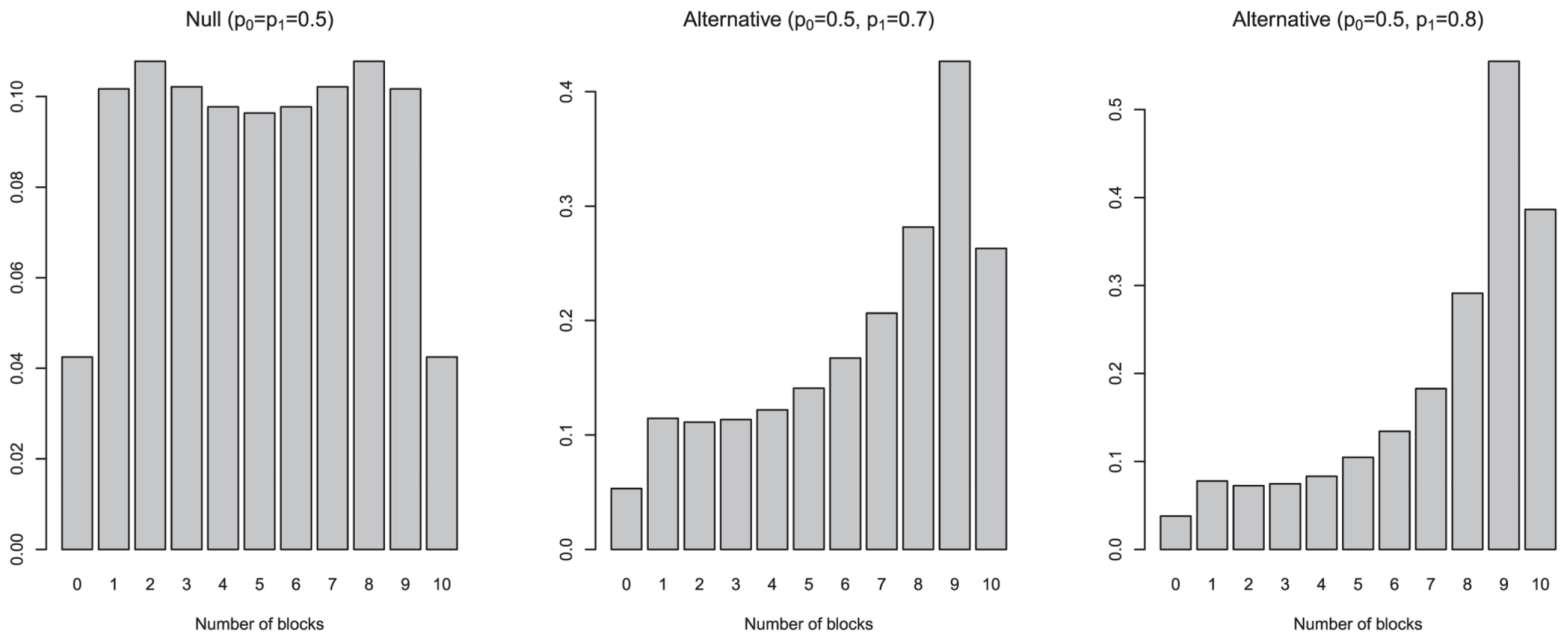
An illustration of the null distribution and two alternative distributions of *Q*, the total number of blocks for which the allocation probability to the experimental arm is greater than 0.5, for *K* = 10, *B = 2* and *n_z_* = 2

The discrete distribution of *Q* under the assumption of no treatment difference is given in Web Appendix D in the online supporting information. Using this distribution we can then find the critical value as the smallest value *c_q_* that satisfies $P\left(Q>c_{q}|p_{0}=p_{1}\right)<\alpha$ and reject the null hypothesis if *Q* > *C_q_* as usual. Note that for larger sample sizes, the calculation of the exact distribution is computationally intensive and it may be more useful in practice to estimate the distribution via MC simulation. The leftmost plot in Figure 1 shows the null distribution of *Q* for a total sample size of 20, split into 10 blocks of size 2, for *n_z_* = 2. The distribution is symmetric about the midpoint of 5 due to the assumption that treatments have equal success probabilities. In this example distribution, the probability of seeing 10 blocks each with allocation probability to the experimental above 0.5 is 0.043. Hence in order to conclude there is evidence to suggest the experimental treatment is superior at the *5%* level, we must observe more than nine allocation probabilities greater than 0.5. For such a test, the power is not adversely affected by the imbalance in treatment groups, in fact the power increases for larger imbalances. A bigger underlying treatment difference gives more skewed allocation probabilities, which means more imbalance between groups (see Figure 1). When performing traditional inference on the outcomes of the trial, for example, using a Fisher exact test, the assumptions required for the validity of the test are violated by the heavy dependencies on the outcomes and sampling direction. Therefore, the increase in power from a larger treatment difference is lessened by the imbalance in treatment groups. When testing using the allocation probabilities as described above, this is not the case. We have constructed a test that has a structural property of the design embedded into it and therefore better aligns to the properties of the experiment underlying it. This highlights the key advantage to the proposed inference approach—no assumptions of the statistical test are violated by an imbalance in treatment groups because the test statistic *Q* is constructed in a framework that supports this imbalance.

## 4 Application

### 4.1 Simulations

In order to compare the use of tests based on allocation probabilities versus those based on success rates, we compare and evaluate the performance with simulated data sets. Since we envisage that the main advantage of using allocation probabilities is to increase the power of the test for superiority, we compare the power for treatments with varying success probabilities in simulated two-arm trials that implement the CARA-FLGI procedure.

We compare the results from the proposed procedure to two other inference methods for trials using the CARA-FLGI. These analyze the results using (a) a Fisher exact test of success rates and (b) a Generalised Linear Model (GLM) with *logit* link function $(\text{logit}\left(\rho_{z}\right)=\beta_{0}+\beta_{1}T\text{,}$ where *ρ_z_* is the success rate for biomarker category *z* and *T* is an indicator variable taking the value 1 if a patient is assigned to the experimental treatment). The robust alternatives of randomization tests are not compared since they have already been shown to be inferior to the Fisher exact test in terms of power (Villar *et al*., 2018) in this class of designs. The only comparison we use to an alternative allocation rule is using ER between the arms and analyzing results using a Fisher exact test on success rates. These trials will have sufficient power, but much smaller patient benefit than those using adaptive randomization and hence are used as a benchmark in terms of power. We have intentionally not included comparisons to alternative CARA designs, since our objective is to improve the power of a particular class of designs by using a novel testing procedure, not to introduce a novel adaptive design. We refer the interested reader to Villar and Rosenberger (2018) for a detailed simulation study comparing the CARA-FLGI to other CARA designs. We also report the percentage of patients on the best treatment, and the total number of observed successes in order to highlight the patient benefit of the CARA-FLGI.

Trials of three different sample sizes are considered, *N* = 40, 80,160. For the CARA-FLGI procedure, a block size of *B* = 2 is used initially with an extension to *B* = 4 and *B* = 8 to assess the impact of block size. For the use of allocation probabilities, the first two blocks’ allocation probabilities are disregarded as a run-in for the CARA-FLGI procedure so that the allocation probabilities used in the testing procedure are meaningful and can be interpreted. In all cases presented here, we use a run-in of two blocks, chosen to maintain power. In practice this run-in can be tailored to suit the expected operating characteristics of the trial, in which case we would recommend at least two and no more than 10% of *K*, the total number of blocks. It must be noted that the run-in is for inference only, the sample size includes those patients in the blocks whose allocation probabilities are disregarded. For each sample size, the number of biomarker categories considered are *n_z_* = 1, 2, 3, 4.

**Figure 2:**
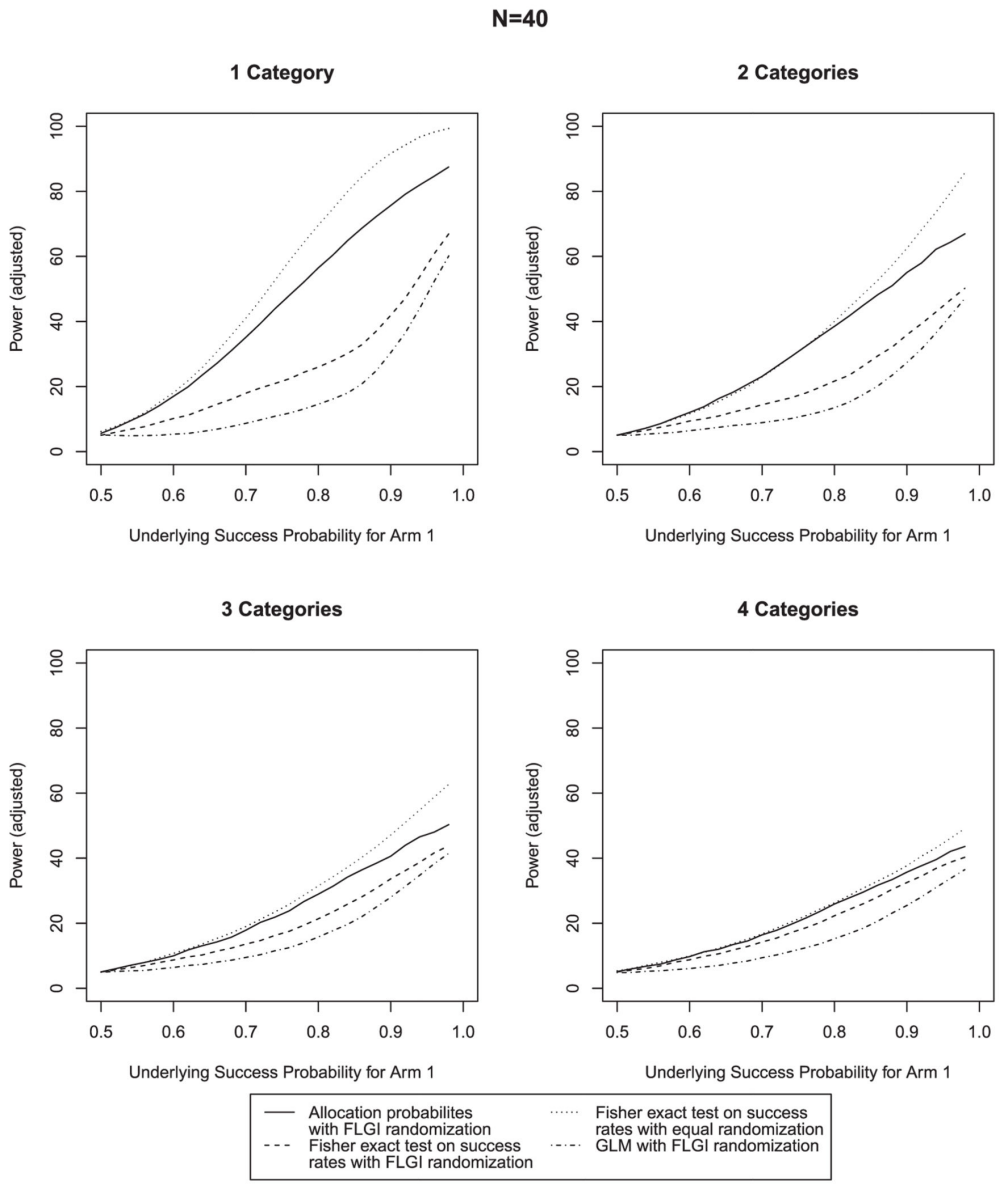
Comparison of power for *N* = 40 & *B* = 2; rejection criteria adjusted for type I error rate

**Figure 3:**
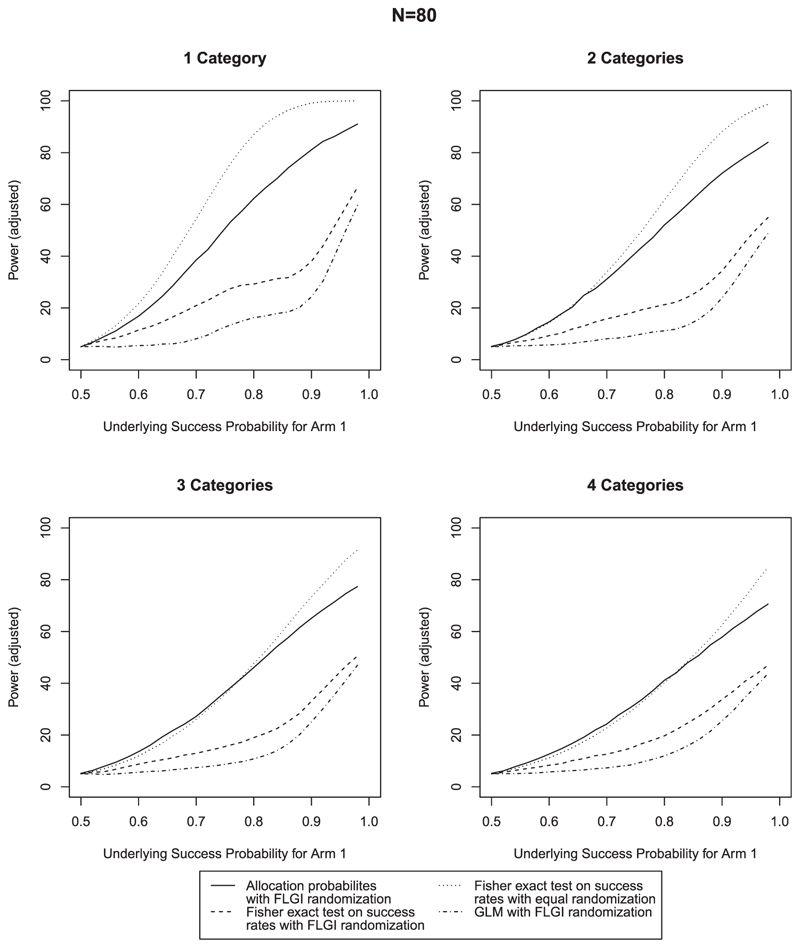
Comparison of power for *N* = 80 & *B* = 2; rejection criteria adjusted for type I error rate

**Figure 4:**
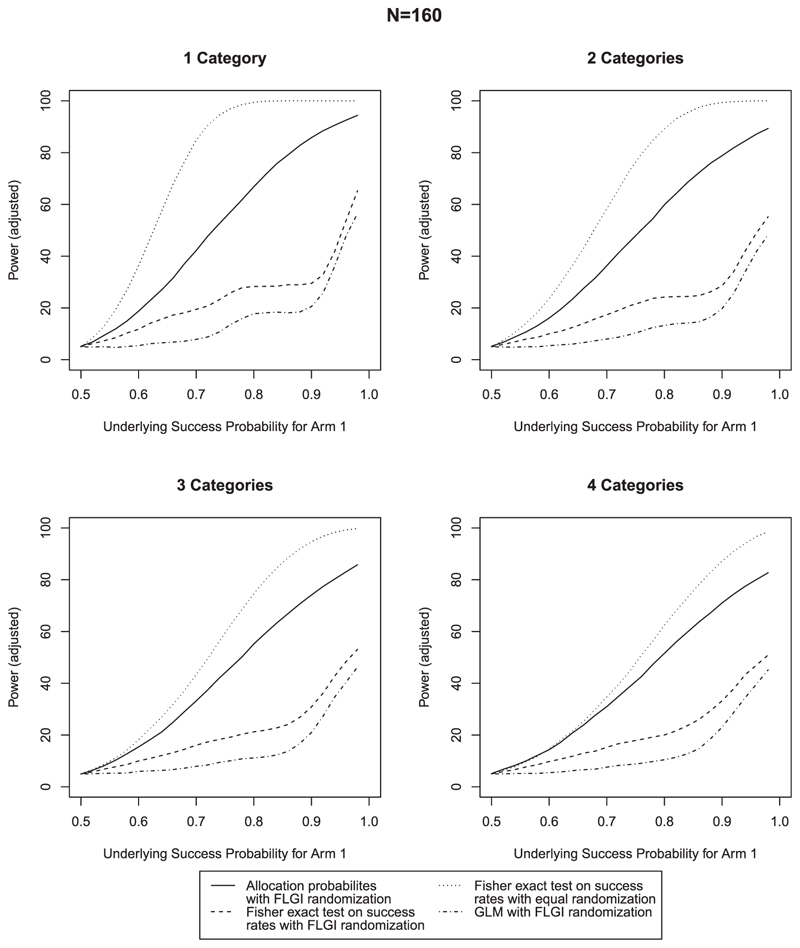
Comparison of power for *N* = 160 & *B* = 2; rejection criteria adjusted for type I error rate

As it is known that the Fisher exact test can lead to a conservative type I error rate (eg, Storer and Kim, 1990), we adjust the rejection criteria in each case to ensure that a 5% type I error rate is observed in the simulations in order for a fair comparison between methods. For the Fisher exact test and GLM, this is implemented by simply adjusting the critical value for rejection. For the test using allocation probabilities, we use a randomized test. Results that are unadjusted for type I error rate are given in Web Appendix A in the online supporting information, which also show that the proposed procedure controls the type I error rate before adjustment in almost all cases. Where this is not the case with smaller *N* and larger *n_z_*, this is due to the null distribution of *Q* having slightly heavier tails. The inflation will be known in advance, and any necessary adjustment can be made. The proposed procedure still shows higher power, even when adjusted for. Figures 2–4 compare the power across the 12 scenarios when type I error is adjusted for. In each case, the success probability of the control treatment is set to 0.5 and the success probability of the experimental treatment increases along the *x*-axis. The most notable characteristic of all of these graphs is that the power curve for the procedure using allocation probabilities of the CARA-FLGI procedure closely follows the curve for the Fisher exact test using success rates in the ER design. At the same time the power of the test based on allocation probability is markedly higher than the Fisher exact test and the logistic model when the CARA-FLGI procedure is used to allocate patients.

The power of the test using allocation probabilities is minimally affected by an increase in the number of categories for the larger sample sizes, whereas the power of the Fisher exact test is adversely affected by an increase in categories. For the scenarios with four biomarker categories, the difference between the Fisher exact test applied to the equal allocation simulations and the use of allocation probabilities applied to the simulations using the CARA-FLGI procedure is at most 20%. Whereas the gain of using allocation probabilities as opposed to the Fisher exact test when the CARA-FLGI is used to allocate patients is up to 40% for the larger sample sizes. The smaller the sample size, the closer the power of the Fisher exact test applied to the equal allocation simulations and the use of allocation probabilities applied to the simulations using the CARA-FLGI procedure. Any difference in power is only noticeable in the case of one biomarker category.

When considering the effect of varying block sizes on the power of the procedure, a comparison between the proposed method using allocation probabilities to test for treatment difference and the Fisher exact test on success rates using FLGI randomization is presented in Figure 4 in Web Appendix A in the online supporting information. For larger numbers of categories, the power of the proposed method is well maintained. However, the power is adversely affected for increasing block size when only one category is considered; there is a clear relationship between both the number of categories and block size.

Although the power of the Fisher exact test on success rates increases for larger block sizes due to the increased balance between treatment groups (and hence lesser patient benefit), it still does not achieve the power of the proposed procedure with *B* = 2. A larger block size may be advantageous in a trial for practical reasons, but both the largest patient benefit and highest power are achieved using the proposed procedure and a smaller block size. If a larger block size is required, in order to maintain power and patient benefit we recommend $B\leq n_{z}+1.$ In relation to this, we also recommend a minimum number of blocks of K≥20.

These results are especially promising when considering the amount of patient benefit that the adaptive randomization offers. Tables 1 and 2 show the percentage of patients that were on the correct treatment across the simulations, for a true underlying treatment difference of 20% and 30%, showing a stark improvement of CARA-FLGI over ER. The tables also show the average total number of successes per trial. Again, there are unsurprisingly far more successes observed for the adaptive design than for ER. For example, for a treatment difference of 20% and *N* = 40, ER gives the average total number of successes of 24. However, with such a small treatment difference, even if all patients were allocated to the superior treatment, this would be 28 and the CARA-FLGI has 26. The modest improvement of patient success is reflective of the scenario and not the CARA-FLGI approach. For a treatment difference of 30%, the average total successes for ER for *N* = 160 is 104, however, if all patients were allocated to the superior treatment, this would be 128 and the CARA-FLGI has 126. The design gives substantial patient benefit over ER and our proposed inference procedure gives comparable power to ER for small block sizes in practice, a clear indication that the proposed procedure has the potential for success.

### 4.2 Illustrative multiarm example

In order to demonstrate the use of FLGI allocation probabilities to test for superiority in a multiarm setting, we use the following trial reported by Attarian *et al*. (2014), which looked at the a combination of baclofen, naltrexone, and sorbitol (PXT3003) in patients with Charcot-Marie-Tooth disease type 1A as an illustrative example. A total of 80 patients were randomized to either the experimental treatment PXT3003 in three different doses, or a control group receiving a placebo. In this trial, ER was used, with 19 patients randomized to the control group, and 21, 21, and 19 patients allocated to the low, intermediate, and high doses of PXT3003, respectively. The aim of the study was to assess both safety and tolerability as well as efficacy, with the measure of safety and tolerability of the total number of adverse events. In the placebo group, a total of 9 out of 19 patients suffered adverse events, whereas this was 5, 7, and 6 in the low, intermediate, and high dose groups, respectively.

We will use this example to simulate how this fourarmed trial with a single biomarker category would have looked using the FLGI procedure with *B* = 2, testing if the allocation probability exceeds 25% across blocks 3 to 40. As there is only one category, we find the critical value, *c_q_* to be 30. In this example, we consider only pairwise comparisons between individual active treatments and control each at full level *α* for simplicity. Should overall control of the family-wise error rate be desired, standard approaches such as a Bonferroni correction or similar adjustment (Simes, 1986) can be applied. Hence we consider the pairwise tests defined by null hypotheses *H*_0*k*_ that there is no treatment effect difference between arm *k* and the control arm, with alternative hypotheses *H*_1*k*_ that active treatment arm *k* is superior to the control arm. We therefore define power in this case as the marginal power, the probability of correctly rejecting null hypothesis *H*_0*k*_ for the treatment *k* with the largest true treatment effect difference from the control.

We will consider three scenarios of varying success rates across the four arms in this illustration. In the first scenario all treatments (including control) have the same success rate of 0.5, while the second scenario uses the estimated success rates from the study itself. The final situation considers a linear dose-response relationship from 0.53 to 0.77 (the lowest to highest observed success rates in the trial) across the four treatment arms.

In 10,000 replications of the trial under the null hypothesis, the type I error rate was well controlled at 5% for the procedure using FLGI allocation probabilities, but was conservative for the Fisher exact test for both allocation schemes. In the scenario, which mimicked the results of the study (some difference between control and active, but hardly any between the different doses), on average 38 patients were allocated to the active treatment with the best underlying success rate and the average total number of successes was 56 compared to the 53 in the original trial. In 34% of the simulations the null hypothesis could be rejected using allocation probabilities compared to 32% to using ER and Fisher exact test. In the final scenario of a linear dose–response a similar trend was observed. Using allocation probabilities to test for superiority led to an increase in power to 41% from the 35% of the Fisher exact test applied to the observed successes in the trial with ER.

## 5 Discussion

RAR can offer patient benefit, but in most cases at the expense of power. In this paper we have introduced a novel inference approach to analyze a clinical trial conducted using an FLGI design, based on a unique perspective on the accumulated information, that does not suffer from decreased power. By using the allocation probabilities generated in the FLGI procedure as opposed to the observed binary outcomes, we address the low power associated with unequal sample sizes inherent in response-adaptive designs.

Although in this paper we have shown promising results for trials implementing the CARA-FLGI rule for patient allocation, it is widely applicable in trials using any FLGI rule. In fact, such is the generality of this novel approach for inference that it can be applied (potentially in some redefined way) to trials using other response-adaptive randomized allocation rules such as the randomized play the winner rule (Wei and Durham, 1978) and similar methods where the allocation probabilities are updated on accumulating patient responses. Our novel approach would not be applicable in cases where a target is not revisited at interims, or is changed by allocations but not by responses, like restricted randomization. We expect the approach to work best when the underlying RAR deviates allocations significantly under the presence of a signal, like the FLGI.

A standard approach in response-adaptive multiarm trials to overcome the low power is to preserve the sample size of the control group (eg, Trippa *et al*., 2012; Villar *etal*., 2015). This is either achieved by starting the trial with an initial period of ER before applying RAR or simply having a fixed allocation to the control arm throughout (although the latter is not applicable in the two arm case, which this novel testing approach is). Both of these do however reduce the patient benefit. One additional advantage of the novel testing approach over such arbitrary rules is that testing on the basis of allocation probabilities yields good power without sacrificing the patient benefit of using RAR for the entire study.

The only valid alternative approach to analyze clinical trials with RAR is randomization-based inference (Simon and Simon, 2011), which is known to be robust but reduce power compared to naive approaches (see Villar *et al*., 2018). Our approach is the first alternative to existing methods to be tailor made to these designs that increases power compared to such naive, and additionally not valid, analysis options.

In this work we have focused our explorations to pairwise testing of two treatment groups. However, RAR is known to perform well for trials with multiple arms (Wason and Trippa, 2014). While we illustrate how a pairwise testing strategy can be applied in this setting further extensions to global tests in multiarmed trials are of interest.

As is commonly the case in RAR, we assume here that the previous block of patients’ responses are available before assignment of the next block; inherent in such designs to increase the patient benefit. However, if desired, both the CARA-FLGI procedure and our novel test may be applied to a setting with delayed responses, subject to minor modifications.

Finally, although focus here has been on superiority of a treatment in a pairwise test, the procedure can be adapted for two-sided tests by considering both tails of the null distribution.

## Supplementary Material

Supplementary File

## Figures and Tables

**Table 1:** Percentage of patients on the correct treatment, and average total observed successes using CARA-FLGI with *B = 2* compared with equal randomization (ER). True treatment difference of 20%

	Patients on correct treatment				
	*CARA -FLGI*				*ER*
	*n_z_* = 1	*n_z_* = 2	*n_z_* = 3	*n_z_* = 4	–
***N* = 40**	77%	71%	67%	65%	50%
***N* = 80**	85%	78%	74%	72%	50%
***N* = 160**	90%	84%	81%	78%	50%
	**Average total successes**				
	***CARA-FLGI***				***ER***
	***n_z_* = 1**	***n_z_* = 2**	***n_z_* = 3**	***n_z_* = 4**	**–**
***N* = 40**	26	26	25	25	24
***N* = 80**	53	52	52	51	48
***N* = 160**	109	107	106	105	96

**Table 2:** Percentage of patients on the correct treatment, and average total observed successes using CARA-FLGI with *B = 2* compared with equal randomization (ER). True treatment difference of 30%

	Patients on correct treatment				
	*CARA—FLGI*				*ER*
	*n_z_* = 1	*n_z_* = 2	*n_z_* = 3	*n_z_* = 4	–
***N* = 40**	86%	80%	76%	73%	50%
***N* = 80**	92%	87%	84%	81%	50%
***N* = 160**	96%	93%	90%	88%	50%
	**Average total successes**				
	***CARA—FLGI***				***ER***
	***n_z_* = 1**	***n_z_* = 2**	***n_z_* = 3**	***n_z_* = 4**	**–**
***N* = 40**	30	30	29	29	26
***N* = 80**	62	61	60	59	52
***N* = 160**	126	124	123	122	104

## Data Availability

Data sharing is not applicable to this paper as no new data were created or analyzed.

## References

[R1] Attarian S, Vallat JM, Magy L, Funalot B, Gonnaud PM, Lacour A (2014). An exploratory randomised double-blind and placebo-controlled phase 2 study of a combination of baclofen, naltrexone and sorbitol (PXT3003) in patients with Charcot-Marie-Tooth disease type 1A. Orphanet Journal of Rare Diseases.

[R2] Gittins JC (1979). Bandit processes and dynamic allocation indices. Journal of the Royal Statistical Society Series B.

[R3] Gittins JC, Glazebrook K, Weber R (2011). Multi-Armed Bandit Allocation Indices.

[R4] Hardwick JP, Stout QF (1991). Bandit strategies for ethical sequential allocation. Computing Science and Statistics.

[R5] He X, Madigan D, Yu B, Wellner J (2019). Statistics at a crossroads: Who is for the challenge? Technical report, The National Science Foundation.

[R6] Hu F, Rosenberger WF, Zhang LX (2006). Asymptotically best response-adaptive randomization procedures. Journal of Statistical Planning and Inference.

[R7] Mozgunov P, Jaki T (2020). An information theoretic approach for selecting arms in clinical trials. Journal of the Royal Statistical Society: Series B.

[R8] Pocock SJ (1979). Allocation of patients to treatment in clinical trials. Biometrics.

[R9] Rosenberger WF, Lachin JM (1993). The use of response-adaptive designs in clinical trials. Controlled Clinical Trials.

[R10] Rosenberger WF, Lachin JM (2016). Randomization in Clinical Trials: Theory and Practice.

[R11] Schulz KF (1996). Randomised trials, human nature, and reporting guidelines. The Lancet.

[R12] Simes RJ (1986). An improved Bonferroni procedure for multiple tests of significance. Biometrika.

[R13] Simon R, Simon NR (2011). Using randomization tests to preserve type I error with response adaptive and covariate adaptive randomization. Statistics and Probability Letters.

[R14] Smith AL, Villar SS (2018). Bayesian adaptive bandit-based designs using the Gittins index for multi-armed trials with normally distributed endpoints. Journal of Applied Statistics.

[R15] Storer BE, Kim C (1990). Exact properties of some exact test statistics for comparing two binomial proportions. Journal of the American Statistical Association.

[R16] Trippa L, Lee EQ, Wen PY, Batchelor TT, Cloughesy T, Parmigiani G (2012). Bayesian adaptive randomized trial design forpatients with recurrent glioblastoma. Journal of Clinical Oncology.

[R17] Vermorel J, Mohri M, Gama J, Camacho R, Brazdil PB, Jorge AM, Torgo L (2005). Machine Learning: ECML.

[R18] Villar SS, Bowden J, Wason J (2015). Multi-armed bandit models for the optimal design of clinical trials: benefits and challenges. Statistical Science.

[R19] Villar SS, Bowden J, Wason J (2018). Response-adaptive designs for binary responses: how to offer patient benefit while being robust to time trends?. Pharmaceutical Statistics.

[R20] Villar SS, Rosenberger WF (2018). Covariate-adjusted response-adaptive randomization for multi-arm clinical trials using a modified forward looking Gittins index rule. Biometrics.

[R21] Villar SS, Wason J, Bowden J (2015). Response-adaptive randomization for multi-arm clinical trials using the forward looking Gittins index rule. Biometrics.

[R22] Wason J, Trippa L (2014). A comparison of Bayesian adaptive randomization and multi-stage designs for multi-arm clinical trials. Statistics in Medicine.

[R23] Wei LJ, Durham S (1978). The randomized play-the-winner rule in medical trials. Journal of the American Statistical Association.

[R24] Williamson SF, Jacko P, Villar SS, Jaki T (2017). A Bayesian adaptive design for clinical trials in rare diseases. Computational Statistics & Data Analysis.

[R25] Williamson SF, Villar SS (2020). A response-adaptive randomization procedure for multi-armed clinical trials with normally distributed outcomes. Biometrics.

