# Supplementary material for: A novel statistical test for treatment differences in clinical trials using a response-adaptive forward-looking Gittins Index Rule
Source: Biometrics. Author manuscript; Available in PMC 2023 Mar 24. (PMC7614356; doi:10.1111/biom.13581)
Supplement: Supplementary File [file EMS144015-supplement-Supplementary_File.zip › Sup_material_revision_final.pdf]

Supporting Information for *A Novel Statistical Test for Treatment Differences in Clinical Trials using a Response Adaptive Forward Looking Gittins Index Rule* by Helen Yvette Barnett, Sofía S Villar, Helena Geys and Thomas Jaki

1 WEB APPENDIX A: Additional Simulation Results

1.1 Web Figure 1

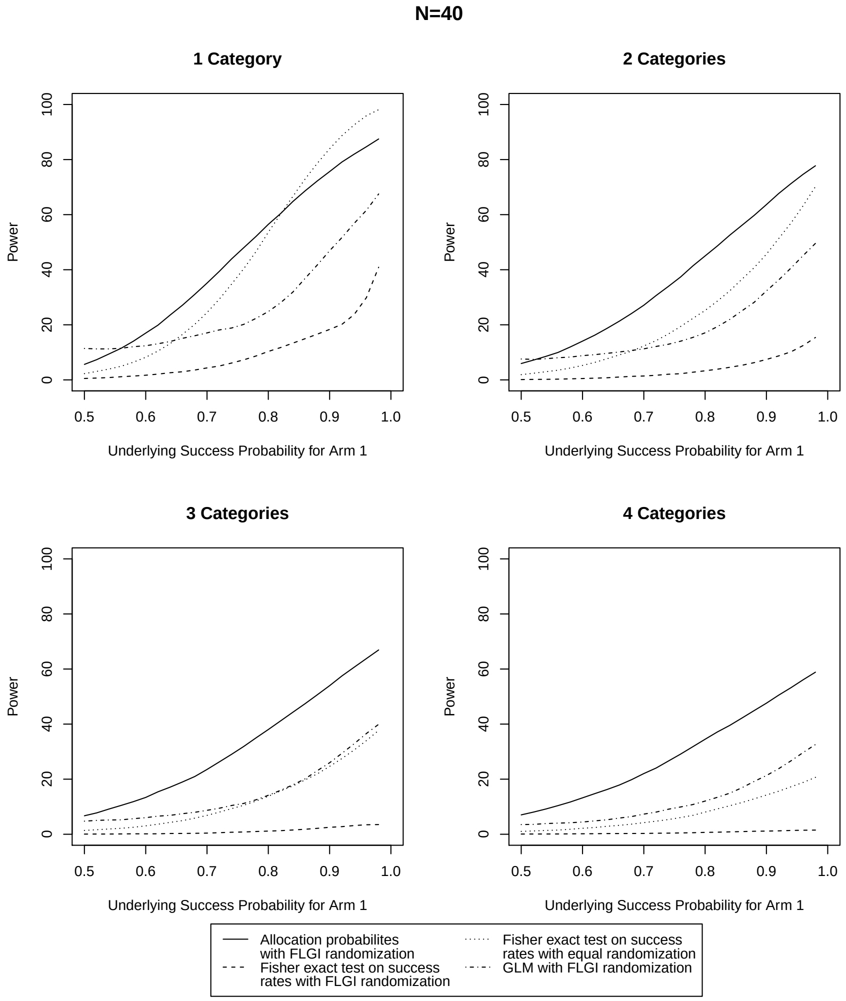

Figure 1: Comparison of power for $N = 40$ & $B = 2$.

1.2 Web Figure 2

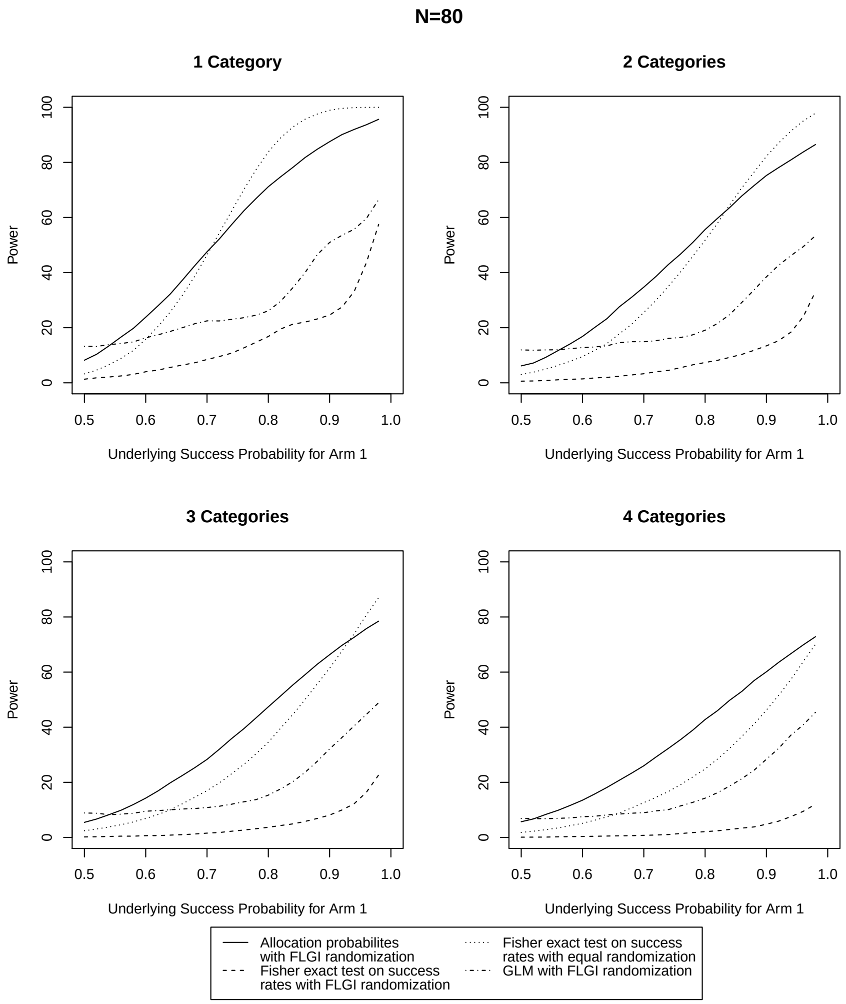

Figure 2: Comparison of power for $N = 80$ & $B = 2$.

1.3 Web Figure 3

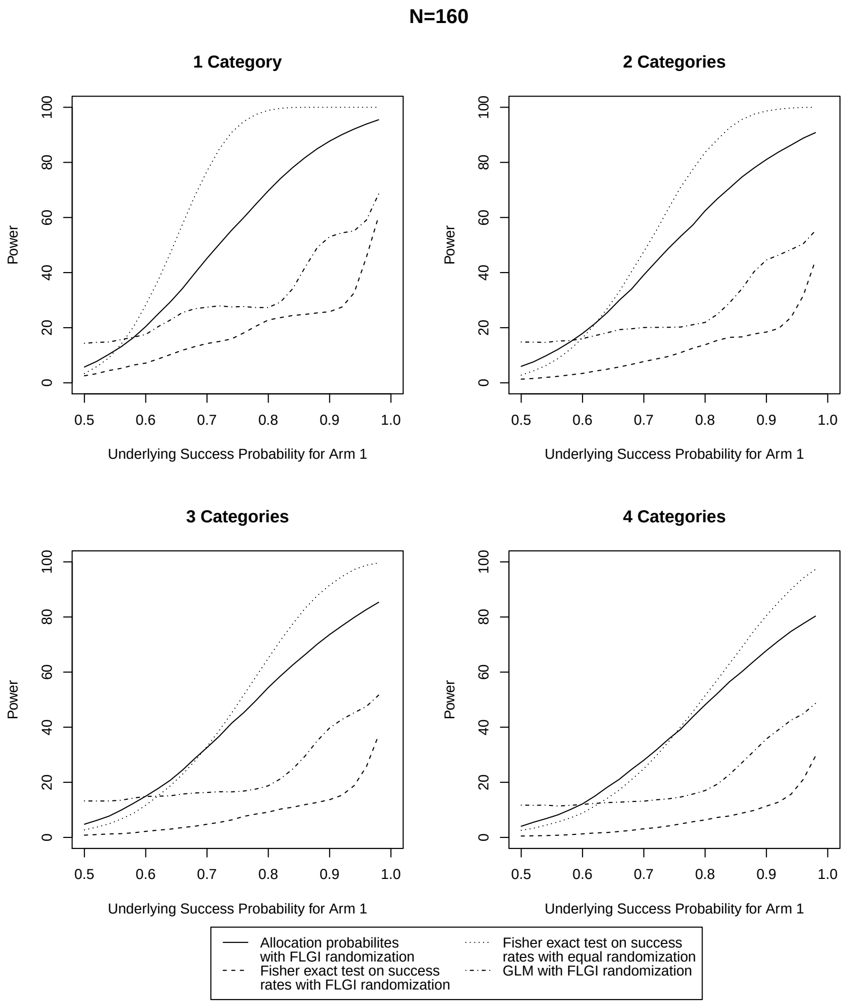

Figure 3: Comparison of power for $N = 160$ & $B = 2$.

1.4 Web Figure 4

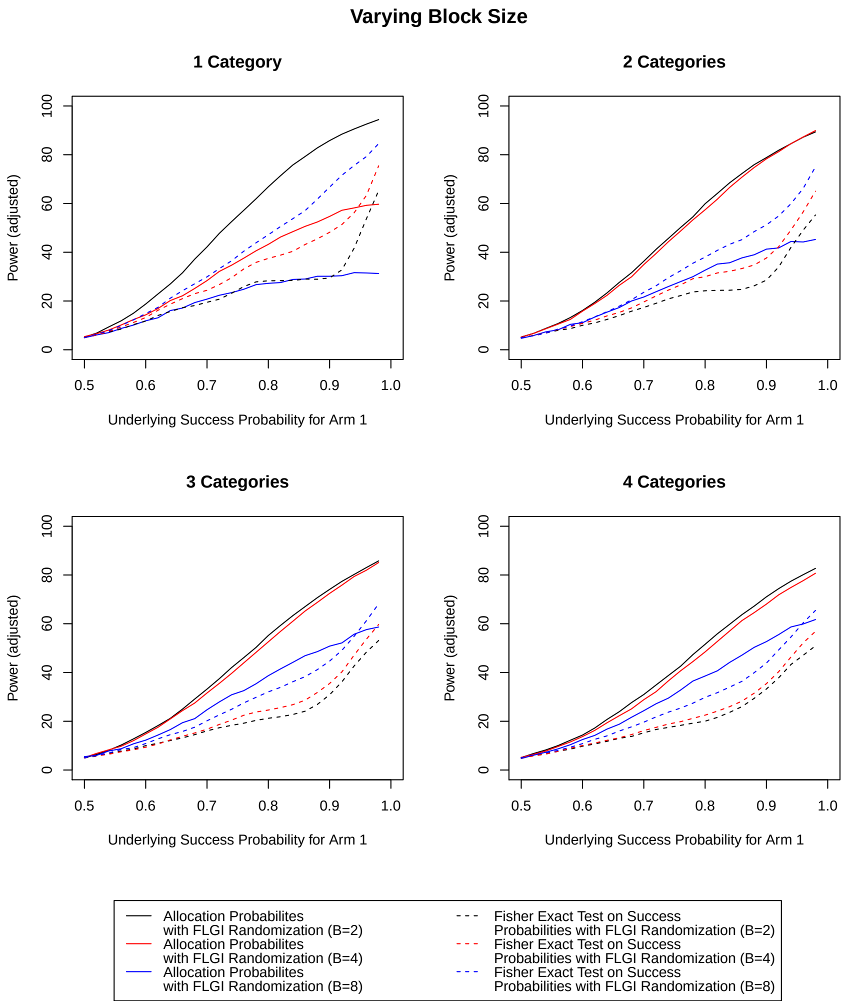

Figure 4: Comparison of power of procedure using allocation probabilities vs Fisher exact test on success rates for $N = 160$ with $B = 2, 4, 8$; rejection criteria adjusted for type I error rate.

2 WEB APPENDIX B: Derivation of Joint Distribution of $X_{j,z}$ & $Y_{j,z}$

This is a discrete distribution described by a $(B+1) \times (B+1)$ matrix of probabilities of observing each of the pairs of values of $X_{j,z}$ and $Y_{j,z}$ from 0 to B.

We first calculate the distribution of $Y_{j,z}$ conditional on $X_{j,z}$ by essentially following a tree diagram starting where the origin is the starting state. This makes decisions using the GI, choosing the arm with the highest GI and the success probability on that arm is the posterior success probability so far on that arm. When the GI is equal, the tree splits into two alternative routes with equal probability, as the algorithm would choose between the arms at random.

We take a "snapshot" after each value of $X_{j,z}$, telling us the probability of each outcome conditional on there being $X_{j,z} = x_{j,z}$ number of patients belonging to category z within the block. To calculate the joint distribution, we multiply this conditional probability by the probability of there being $x_{j,z}$ number of patients in category z, following the Binomial distribution previously introduced.

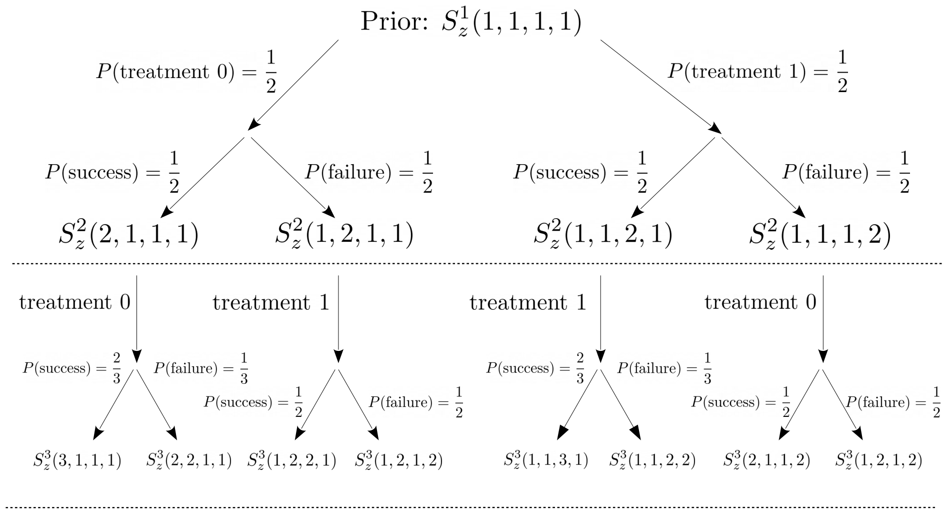

Figure 5: An illustration of tree diagram used in calculation of distribution of $p_{\text{alpro, z}}$, with $B = 2$.

As an illustration, Figure 5 shows how this is calculated with a block size of 2. The probabilities on the tree are all conditional on $X_{j,z} = 2$, implying that of the block of two patients, both are in the given biomarker category z. Success and failure probabilities are the posterior probabilities for a given treatment . The dashed line indicates the level at which to take each "snapshot". For example the probability of having two patients in category z allocated to treatment 1 conditional on two patients being in category z is the sum of following the two branches that result in state $S_z^3(1,1,3,1)$ and $S_z^3(1,1,2,2)$. These branches have probability

$\frac{1}{2} \times \frac{1}{2} \times \frac{2}{3} = \frac{1}{6}$ and $\frac{1}{2} \times \frac{1}{2} \times \frac{1}{3} = \frac{1}{12}$ respectively, and hence the $\mathbb{P}(Y_{j,z} = 2|X_{j,z} = 2) = \frac{1}{4}$ and therefore $\mathbb{P}(Y_{j,z} = 2 \text{ \& } X_{j,z} = 2) = \mathbb{P}(Y_{j,z} = 2|X_{j,z} = 2)\mathbb{P}(X_{j,z} = 2) = \frac{1}{4n_z^2}$.

In the general case, the joint distribution of $Y_{j,z}$ and $X_{j,z}$ is given as:

$$\begin{aligned}
\mathbb{P}(Y_{j,z} = y_{j,z} \text{ \& } X_{j,z} = x_{j,z}) &= \binom{B}{x_{j,z}} \left(\frac{1}{n_z}\right)^{x_{j,z}} \left(\frac{1-n_z}{n_z}\right)^{B-x_{j,z}} \times \\
&\qquad \mathbb{P}(Y_{j,z} = y_{j,z}|X_{j,z} = x_{j,z}) \\
&= \binom{B}{x_{j,z}} \left(\frac{1}{n_z}\right)^{x_{j,z}} \left(\frac{1-n_z}{n_z}\right)^{B-x_{j,z}} \times \\
&\qquad \sum_{\mathcal{S}^i_{z,x_{j,z},y_{j,z}}} \mathbb{P}(S^i_z(s^i_{0,z}, f^i_{0,z}, s^i_{1,z}, f^i_{1,z})),
\end{aligned}$$

where $\mathcal{S}^i_{z,x_{j,z},y_{j,z}}$ is the set of all states $S^i_z(s^i_{0,z}, f^i_{0,z}, s^i_{1,z}, f^i_{1,z})$ such that $s^i_{0,z} + f^i_{0,z} + s^i_{1,z} + f^i_{1,z} - (s^1_{0,z} + f^1_{0,z} + s^1_{1,z} + f^1_{1,z}) = x_{j,z}$ and $s^i_{1,z} + f^i_{1,z} - (s^1_{1,z} + f^1_{1,z}) = y_{j,z}$.

For the generic state $S^i_z(s^i_{0,z}, f^i_{0,z}, s^i_{1,z}, f^i_{1,z})$, let $\mathfrak{S}^{i-1}_z$ be the set of four possible different states that may precede this state. Then

$$\mathbb{P}(S^i_z(s^i_{0,z}, f^i_{0,z}, s^i_{1,z}, f^i_{1,z})) = \sum_{S^{i-1}_z \in \mathfrak{S}^{i-1}_z} \mathbb{P}(S^i_z(s^i_{0,z}, f^i_{0,z}, s^i_{1,z}, f^i_{1,z})|S^{i-1}_z)\mathbb{P}(S^{i-1}_z).$$

Let κ^{i-1}_z equal 1 when the previous patient in category z was allocated to the experimental treatment and 0 if allocated to the control. Then let $T^{i-1}_{\kappa^{i-1}_z,z}$ equal 1 if the outcome on treatment κ^{i-1}_z was a success and 0 if it was a failure, so that for example

$$\mathbb{P}(\kappa^{i-1}_z = 0, T^{i-1}_{0,z} = 1) = \mathbb{P}(S^i_z(s^i_{0,z}, f^i_{0,z}, s^i_{1,z}, f^i_{1,z})|S^{i-1}_z(s^i_{0,z} - 1, f^i_{0,z}, s^i_{1,z}, f^i_{1,z})).$$

This is calculated for all four possible states that could precede this states by

$$\mathbb{P}(\kappa^{i-1}_z = \kappa, T^{i-1}_{\kappa,z} = t) = \begin{cases} 0 & \text{if } GI(s^i_{\kappa,z}-t, f^i_{\kappa,z}-(1-t)) < GI(s^i_{1-\kappa,z}, f^i_{1-\kappa,z}) \\ \frac{ts^i_{\kappa,z}+(1-t)f^i_{\kappa^{i-1},z}-1}{2(s^i_{\kappa,z}+fi_{\kappa,z}-1)} & \text{if } GI(s^i_{\kappa,z}-t, f^i_{\kappa,z}-(1-t)) = GI(s^i_{1-\kappa,z}, f^i_{1-\kappa,z}) \\ \frac{ts^i_{\kappa,z}+(1-t)f^i_{\kappa,z}-1}{(s^i_{\kappa,z}+f^i_{\kappa,z}-1)} & \text{if } GI(s^i_{\kappa,z}-t, f^i_{\kappa,z}-(1-t)) > GI(s^i_{1-\kappa,z}, f^i_{1-\kappa,z}), \end{cases}$$

where we classify the three cases that may occur in the previous state. The first is that the Gittins Index of the state for the chosen treatment allocation κ is less than the treatment not chosen $1 - \kappa$, in which case the probability is 0 since this is not a plausible scenario. The second case is that the two treatments had equal Gittins Index, in which case the probability is the product of the probability of allocating the chosen treatment (1/2) and the success (or failure for $T^{i-1}_{\kappa,z} = 0$) probability (which is the posterior success (or failure) probability at the current state). The final case is that where the Gittins Index is greater for the allocated treatment than the treatment not allocated. In this case, the probability is simply the posterior probability of success (or failure for $T^{i-1}_{\kappa,z} = 0$)

Each of the probabilities of the states S^{i-1}_z can then be recursively calculated in the same way.

3 WEB APPENDIX C: Proofs

3.1 Proof of Lemma 3.1

Proof. Let $p^{t_1}_{\text{alpro, z}}$ be the allocation probability to the experimental treatment and $p^{t_0}_{\text{alpro, z}}$ be the allocation probability to the control treatment. By symmetry, the distribution of $p^{t_1}_{\text{alpro, z}}$ at $S^i_z(s^i_{0,z}, f^i_{0,z}, s^i_{1,z}, f^i_{1,z})$ is the same as the distribution of the $p^{t_0}_{\text{alpro, z}}$ at $S^i_z(s^i_{1,z}, f^i_{1,z}, s^i_{0,z}, f^i_{0,z})$, regardless of assumptions on treatment difference. Since from any state $p^{t_1}_{\text{alpro, z}} = 1 - p^{t_0}_{\text{alpro, z}}$, we may take, for $0 \leq c \leq 1$

$$
\begin{aligned}
F^{t_1}_{s^i_{0,z} f^i_{0,z} s^i_{1,z} f^i_{1,z}}(c) &= F^{t_0}_{s^i_{1,z} f^i_{1,z} s^i_{0,z} f^i_{0,z}}(c) \\
&= \mathbb{P}(p^{t_0}_{\text{alpro},s^i_{1,z} f^i_{1,z} s^i_{0,z} f^i_{0,z}} < c) \\
&= \mathbb{P}(1 - p^{t_1}_{\text{alpro},s^i_{1,z} f^i_{1,z} s^i_{0,z} f^i_{0,z}} < c) \\
&= \mathbb{P}(1 - c < p^{t_1}_{\text{alpro},s^i_{1,z} f^i_{1,z} s^i_{0,z} f^i_{0,z}}) \\
&= 1 - \mathbb{P}(p^{t_1}_{\text{alpro},s^i_{1,z} f^i_{1,z} s^i_{0,z} f^i_{0,z}} < 1 - c) \\
&= 1 - F^{t_1}_{s^i_{1,z} f^i_{1,z} s^i_{0,z} f^i_{0,z}}(1 - c).
\end{aligned}
$$

Therefore $F^{t_1}_{s^i_{0,z} f^i_{0,z} s^i_{1,z} f^i_{1,z}}(0.5) = 1 - F^{t_1}_{s^i_{1,z} f^i_{1,z} s^i_{0,z} f^i_{0,z}}(0.5)$. □

3.2 Proof of Lemma 3.2

Proof. Since this Lemma is for a necessary and sufficient condition, we split the proof into two distinct parts. Part (a) is the proof that consistently higher allocation probabilities for arm 1 implies $p_1 - p_0 > 0$. Part (b) is the proof that $p_1 - p_0 > 0$ implies consistently higher allocation probabilities for arm 1.

Part (a):

We intend to prove this in two steps. First, we show that consistently higher allocation probabilities for one arm indicates a true treatment difference. We prove this by showing the contrapositive; that no true treatment difference implies the allocation probability is equally likely to be above or below 0.5 within a trial of size N.

The distribution of $p^{t_1}_{\text{alpro, z}}$ at the beginning of block k is given by the mixture distribution in equation (4) in the main paper. Consider the set of all states Z as pairs of "mirror" states and individual "symmetrical" states (where $s^i_{0,z} = s^i_{1,z}$ & $f^i_{0,z} = f^i_{1,z}$). Take first the "symmetrical" states, noting that $GI(s^i_{0,z}, f^i_{0,z}) = GI(s^i_{1,z}, f^i_{1,z})$. We therefore have $\mu_{y,z} = \frac{\mu_{x,z}}{2}$, since within each block of the Monte-Carlo runs, we expect to see equal allocations between treatments. Substituting this into F, we see that $F(0.5) = 0.5$, indicating that the $p_{\text{alpro, z}}$ for both treatments is equally likely to be above and below 0.5. Likewise this is true for the exact calculations by **?**.

Now consider the pairs of "mirror" states. As a consequence of lemma 3.1, the distribution of the allocation probabilities to the experimental treatment from these pairs of states are

reflections about $p_{\text{alpro, z}} = 0.5$. Under the assumption that $p_1 - p_0 = 0$, these states are also equally probable. Hence, when the weighted sum of distributions for both "mirror" and "symmetrical" states is calculated in the following Section (equation (4) in the main paper), the overall effect is that allocation probabilities above and below 0.5 are equally likely.

Now that we have established that consistently higher allocation probabilities for one arm imply $p_1 \neq p_0$, we consider the inequality relating p_1 and p_0 when a given arm has consistently higher allocation probabilities. This is a strict inequality, either $p_1 > p_0$ or $p_1 < p_0$ since in the first part of this proof we showed $p_1 \neq p_0$.

For the second part of this proof, we show that consistently higher allocation probabilities for arm 1 imply $p_1 > p_0$. We assume for contradiction the negation of this statement, that we have consistently higher allocation probabilities for arm 1 and $p_1 < p_0$. Since the allocation probabilities for arm 1 are consistently higher, more patients in the trial are allocated to treatment 1 than treatment 0. However, because the true success probability of treatment 1 is lower than treatment 0, this trial design has fewer expected success than a trial design where arm 0 has consistently higher allocation probabilities and hence more patients are allocated to treatment 0 than treatment 1. Since the FLGI procedure is near optimal in maximising expected successes, we have a contradiction in that there clearly exists another design with a much higher level of expected successes. Hence consistently higher allocation probabilities for arm 1 do indeed imply $p_1 > p_0$.

Part (b):

As the number of patients in the trial, N, gets very large, the posterior success probabilities for each of the two treatments will tend to the true success probabilities for those treatments. Therefore, for any finite block size B and true difference in success probabilities $p_1 - p_0 > 0$, there exists $\epsilon > 0$ such that

$$\mathbb{P}\left(\left|\frac{s_{1,z}^N}{s_{1,z}^N + f_{1,z}^N} - \frac{s_{0,z}^N}{s_{0,z}^N + f_{0,z}^N} - (p_1 - p_0)\right| < \epsilon\right) \to 1 \quad \text{as} \quad N \to \infty.$$

Hence for all $0 < m \leq 1$, when $p_1 - p_0 \geq m$ there exists $0 < \epsilon < m$ such that

$$\mathbb{P}\left(\frac{s_{1,z}^N}{s_{1,z}^N + f_{1,z}^N} - \frac{s_{0,z}^N}{s_{0,z}^N + f_{0,z}^N} \geq m - \epsilon\right) \to 1 \quad \text{as} \quad N \to \infty.$$

Therefore for all $b = 0, \dots, B$

$$\mathbb{P}\left(GI(s_{1,z}^N, f_{1,z}^N + b) > GI(s_{0,z}^N, f_{0,z}^N)\right) \to 1 \quad N \to \infty.$$

In practice this means that in the state $S_z^N(s_{0,z}^N, f_{0,z}^N, s_{1,z}^N, f_{1,z}^N)$, no matter how unlikely the outcome (success/failure) and category classification (all/no patients in given category), every patient in the following block in the Monte-Carlo simulations used to calculate the allocation probability will be allocated to the experimental treatment. Consequently, in equation (1) in the main paper, we have $X_{j,z} = Y_{j,z}$ for all $j = 1, \dots, n$ and hence $p_{\text{alpro, z}}^{t_1} = 1$.

In fact, as long as $N >> B$, the state $S_z^N(s_{0,z}^N, f_{0,z}^N, s_{1,z}^N, f_{1,z}^N)$ with $GI(s_{0,z}^i, f_{0,z}^i) < GI(s_{1,z}^i, f_{1,z}^i)$ is more likely than its "mirror" state $S_z^N(s_{1,z}^N, f_{1,z}^N, s_{0,z}^N, f_{0,z}^N)$ when $p_1 - p_0 > m$. Further, as

a consequence of lemma 3.1, the distribution of the allocation probabilities to the experimental treatment from these states are reflections about $p_{\text{alpro, z}} = 0.5$. Therefore when the weighted sum of all potential distributions is calculated in the following Section (equation (4) in the main paper), the overall effect is that $\mathbb{P}(p_{\text{alpro, z}} > 0.5) > 0.5$.

□

4 WEB APPENDIX D: Specification of null distribution

For a block of size B, there are $\eta = 0, 1, \ldots B$ potential patients in the block in a given biomarker category z. In general, the number of ways to put r objects in R boxes is $\binom{r+R-1}{R-1}$. For a given η, we have η objects (patients), to put in 4 boxes ($s_{0,z}$, $f_{0,z}$, $s_{1,z}$, $f_{1,z}$). Therefore the number of potential states at the end of block 1, M_1, is the sum over the number of potential patients within the category and block, of the number of potential states at the end of block 1:

$$M_1 = \sum_{\eta=0}^{B} \binom{\eta+3}{3}.$$

Thus for the next blocks, the number of potential states at the end of block k is

$$M_k = \sum_{\eta=0}^{kB} \binom{\eta+3}{3}.$$

Define f_ζ as the distribution of allocation probabilities from a given state ζ, as described in equation (3) in the main paper. Let c_p be the previous allocation probability, so in the following when integrating over [0.5,1], it indicates we are conditioning on a previous $\alpha_k = 1$ whereas integrating over [0,0.5] indicates we are conditioning on a previous $\alpha_k = 0$.

Let $\mathbb{P}(\zeta|c_p)$ be the probability of being in state ζ conditional on the previous allocation probability. That is, the allocation probability that was calculated at the beginning of the previous block. This can be calculated in a similar manner as shown in Figure 5. A tree starting at each potential state the previous block could have ended in is needed, following the possible allocations and success/failure along the branches to find the conditional probability. Again including a multiplicative factor of the relevant binomial probability at each dotted line to calculate the joint probability. Two major differences however, are that patient allocation is random with probability determined by the previous calculated allocation probability c_p as opposed to the deterministic GI rule, and the success is determined by a pre-specified equal success probability for both treatments as opposed to posterior probability.

With an uninformative prior, for the first block we simply have the case that $\mathbb{P}(\alpha_1 = 0) = \mathbb{P}(\alpha_1 = 1) = 0.5$. Then for each potential state ζ that the first block could end in, the joint probability of the first block ending in that state ζ and the first allocation probability being less than 0.5 is

$$\mathbb{P}(\zeta, \alpha_1 = 0) = \int_{c_p=0}^{c_p=0.5} \mathbb{P}(\zeta|prior, c_p) f_{prior}(c_p) dc_p. \tag{1}$$

We define the set of all states ζ such that $\mathbb{P}(\zeta, \alpha_1 = 0) \neq 0$ as Z_0, and the set of all states ζ such that $\mathbb{P}(\zeta, \alpha_1 = 1) \neq 0$ as Z_1

These satisfy the following

$$\mathbb{P}(\alpha_1 = 0) = \sum_{\zeta \in Z_0} \int_{c_p=0}^{c_p=0.5} \mathbb{P}(\zeta|prior, c_p) f_{prior}(c_p) dc_p = 0.5, \tag{2}$$

and

$$\mathbb{P}(\alpha_1 = 1) = \sum_{\zeta \in Z_1} \int_{c_p=0.5}^{c_p=1} \mathbb{P}(\zeta | prior, c_p) f_{prior}(c_p) dc_p = 0.5. \tag{3}$$

Then consider the second block. Again, the joint probability of the second block ending in state ζ and the first two allocation probabilities being less than 0.5 is

$$\mathbb{P}(\zeta, \alpha_1 = 0, \alpha_2 = 0) = \sum_{\zeta_p \in Z_0} \mathbb{P}(\zeta_p, \alpha_1 = 0) \int_{c_p=0}^{c_p=0.5} \mathbb{P}(\zeta | \zeta_p, c_p) f_{\zeta_p}(c_p) dc_p. \tag{4}$$

We define the set of all states ζ such that $\mathbb{P}(\zeta, \alpha_1 = 0, \alpha_2 = 0) \neq 0$ as Z_{00}, and in general $Z_{\alpha_1 \alpha_2}$ is the set of all states ζ that have a non-zero probability of occurrence at the end of block 2 when the first two allocation probabilities are identified by α_1 and α_2.

So that we can for example calculate the probability of the first two allocation probabilities being less than 0.5 by summing over the set Z_{00}

$$\mathbb{P}(\alpha_1 = 0, \alpha_2 = 0) = \sum_{\zeta \in Z_{00}} \mathbb{P}(\zeta, \alpha_1 = 0, \alpha_2 = 0) \tag{5}$$

The calculations for subsequent blocks follow on, for example if we wish to calculate the probability of the sequence $\alpha_1 = 0, \alpha_2 = 0, \alpha_3 = 1$, we first calculate

$$\mathbb{P}(\zeta, \alpha_1 = 0, \alpha_2 = 0, \alpha_3 = 1) = \sum_{\zeta_p \in Z_{00}} \mathbb{P}(\zeta_p, \alpha_1 = 0, \alpha_2 = 0) \int_{c_p=0.5}^{c_p=1} \mathbb{P}(\zeta | \zeta_p, c_p) f_{\zeta_p}(c_p) dc_p. \tag{6}$$

Then sum over the set Z_{001}

$$\mathbb{P}(\alpha_1 = 0, \alpha_2 = 0, \alpha_3 = 1) = \sum_{\zeta \in Z_{001}} \mathbb{P}(\zeta, \alpha_1 = 0, \alpha_2 = 0, \alpha_3 = 1). \tag{7}$$

However, these calculations are extremely computationally intensive once larger numbers of blocks are considered, and hence by the linearity of expectation (since $\mathbb{P}(\zeta | \zeta_p, c_p)$ is linear in c_p), we use the following. It is no longer needed to integrate over c_p, one can instead find the expectation of c_p with respect to the distribution f_{ζ_p}, $\mathbb{E}_{f_{\zeta_p}}[c_p | c_p < 0.5]$ conditional on $c_p < 0.5$ and rewrite (4) as:

$$\mathbb{P}(\zeta, \alpha_1 = 0, \alpha_2 = 0) = \sum_{\zeta_p \in Z_0} \mathbb{P}(\zeta_p, \alpha_1 = 0) \mathbb{P}(\zeta | \zeta_p, c_p = \mathbb{E}_{f_{\zeta_p}}[c_p | c_p < 0.5]). \tag{8}$$

Due to the nature of the procedure, the ordering of the α_k is not exchangeable. For example $\mathbb{P}(\alpha_1 = 1, \alpha_2 = 1, \alpha_3 = 0)$ is not necessarily the same as $\mathbb{P}(\alpha_1 = 1, \alpha_2 = 0, \alpha_3 = 1)$, even though for both sequences $\sum_{k=1}^{3} \alpha_k = 2$. To calculate the probability of observing a given value for $\sum_{k=1}^{K} \alpha_k$, the probability of each sequence with this corresponding value of $\sum_{k=1}^{K} \alpha_k$ is summed over, since they are mutually exclusive.

From this discrete null distribution, we can find a critical value for 5% type I error in the test.
